# The influence of home isolation during COVID-19 on the physical fitness development of college students: a study utilizing repeated measures analysis of variance

**DOI:** 10.1186/s12889-023-16772-8

**Published:** 2023-11-07

**Authors:** Chang Jie, Sun Xugui, Zhang Min, Zhu Ergang, Wang Hongwu, Sun Jun

**Affiliations:** 1 Department of Medical Information, Wannan Medical College, Wuhu, China; 2 Department of Public Foundation, Wannan Medical College, Wuhu, China; 3 Physical Education Institute, Chizhou University, Chizhou, China; 4 Public Health Science and Engineering College, Tianjin University of Traditional Chinese Medicine, Tianjin, China

**Keywords:** COVID-19, Student, Physical fitness

## Abstract

**Background:**

Research on the impact of COVID-19-induced home isolation on the physical fitness of college students is limited. This study aims to compare and analyze the physical fitness test scores of college students in two groups: those who experienced home isolation and those who did not, over three consecutive years after enrolment, to investigate the effects of home isolation on the physical fitness development of Chinese college students.

**Methods:**

This comparative study included two longitudinal surveys conducted among medical college students. The participants were divided into an experimental group and a control group. The physical fitness indicators measured included body mass index (BMI), vital capacity (VC), 50-metre run, sit-and-reach, standing long jump, 1000/800-metre runs (males/females), pull-ups (males) and sit-ups (females). Repeated measures analysis of variance (ANOVA) was employed, and the Greenhouse-Geisser correction was applied when Mauchly’s assumption of sphericity was violated. Pairwise comparisons were conducted using the Bonferroni method.

**Results:**

A total of 6580 students participated in the study, with 3360 students (1490 males, 1870 females) enrolled in 2019 as the experimental group and 3220 students (1326 males, 1894 females) enrolled in 2017 as the control group. All participants completed the physical fitness tests for three consecutive years. The results showed that the experimental group exhibited decreased performance in the 1000-metre and 800-metre runs, and improved performance in the sit-and-reach test. After the end of home isolation, there was an improvement in the performance of the 1000-metre run and 800-metre run, while no significant differences were observed in the trends of the other tested indicators.

**Conclusion:**

The findings of this study indicate that the home isolation environment during COVID-19 had a significant impact on the physical fitness of college students, specifically in terms of endurance and flexibility qualities, as well as male BMI. To better prepare for future public health emergencies and mitigate the effects of isolation, teaching students endurance exercises that can be performed at home should be prioritized. Furthermore, physical education programs should be improved to enhance student flexibility.

## Background

The emergence of the coronavirus disease 2019 (COVID-19) outbreak at the end of 2019 significantly disrupted regular teaching activities in schools globally [1]. However, ensuring the continuous progress of these educational activities remains a vital endeavor for nurturing the development of the younger generation. In response to curbing the spread of COVID-19 and safeguarding students’ health, the Chinese government implemented measures such as delaying the commencement of the academic year and mandating students to remain at home [2]. It was only in September 2020 that universities gradually resumed in-person teaching, accompanied by stringent management and preventive protocols including restricted campus access, mask mandates, physical distancing, improved hygiene practices, enhanced ventilation, and limitations on large gatherings [3].

While these preventive measures have effectively curbed the further spread of COVID-19 [4], they unavoidably disrupted the typical lives of college students. Research has highlighted that the home isolation and restricted activities have led to notable changes in college students’ lifestyles and dietary habits [5]. Particularly, a considerable decrease in physical activity levels and an increase in sedentary behaviors among college students have been observed [6, 7].

These changes have resulted in adverse effects on their physical health, elevating the risk of various health concerns including COVID-19 infection, muscle atrophy, decreased metabolism, obesity, cardiovascular diseases, and other related conditions [8]. Additionally, the constraints on activities have not only affected physical health but have also negatively impacted the mental well-being of college students, evident through an increase in depression and anxiety symptoms during the pandemic [9].

In the context of recent research, our study categorizes studies on the impact of isolation environments on physical fitness into two primary groups. The first category examines changes in physical fitness within the same population before and after isolation [10, 11]. The second category involves comparing changes in physical fitness before and after isolation among different populations [12, 13]. Despite the shared methodologies in these studies, a direct causal link between changes in physical fitness and the pandemic remains elusive.

To foster a more scientifically robust analysis of the effects of the isolation environment, our study was initiated in 2017. Employing a longitudinal comparative approach, we meticulously contrasted the group of students who commenced their studies in 2019 and experienced the epidemic’s impact with the group of students who began in 2017 and remained unaffected by the pandemic’s upheaval. This methodological choice ensures a stringent evaluation of the influence of the epidemic-related isolation environment on physical fitness.

Given this context, our primary research objective is to meticulously compare and analyze the physical fitness test results of university students over three consecutive years under both the epidemic-related isolation setting and the non-affected conditions. Through this comparison, we aim to gain insights into the precise impact of home isolation on the development of university students’ physical fitness. In addition to the primary goal, our secondary objective is to provide strategic directions and recommendations to enhance the physical fitness of university students during periods of home isolation. This proactive approach seeks to equip students with strategies for coping with unforeseen public health crises in the future.

Building on previous research outcomes, we hypothesize that the home isolation environment may exert an adverse impact on the progression of university students’ physical fitness. As such, our study tackles a critical concern and contributes valuable insights to address both present and future challenges.

## Methods

**Fig. 1:**
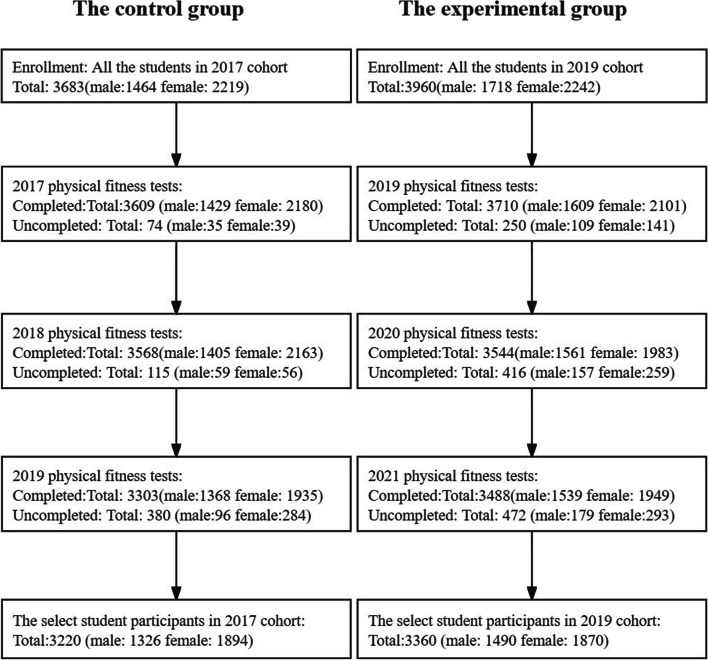
Comprehensive information on selected student participants in control group (2017) and experimental group (2019) across three years after enrollment

A continuous three-year physical fitness test was conducted on two groups (the control group and the experimental group) of students in Wannan Medical College after their enrolment. The control group was enrolled in the college 2017, while the experimental group was enrolled in 2019. The decision to designate students enrolled in 2017 as the control group was made to ensure that this cohort remained unaffected by the COVID-19 pandemic during the three-year observation period. The participants’ ages ranged from 17 to 22 years. Annually, there were students who could not complete the physical fitness test due to factors like illness, military service, or leave of absence. Figure 1 displays comprehensive information pertaining to the selected student participants in both the control group (2017) and the experimental group (2019) across the first to third years subsequent to their enrollment. The research protocol was approved by the Research Ethics Review Committee of Wannan Medical College (Ref No: 2022-093), and informed consent was obtained from all participating students prior to commencement of the study.

The physical fitness test data for the control group were collected during the years 2017 to 2019, encompassing the first, second, and third years of the control group. For the experimental group, the data were collected from 2019 to 2021, covering the corresponding first, second, and third years of the experimental group. It is important to highlight that even though both the control group and the experimental group were observed in 2019, the tested data of the control group in 2019 pertains to the third year’s data, while the tested data of the experimental group in 2019 pertains to the first year’s data. The tests included body mass index (BMI), vital capacity (VC) measurement, 50-metre run, sit-and-reach, standing long jump, 1000/800-metre runs, pull-ups (males) and sit-ups (females). Following [14], the detailed measurements are as follows:

- Body Mass Index (BMI): BMI was calculated using the standard formula (weight [*kg*] divided by height [$m^2$]). Based on the World Health Organization standards, BMI was categorized into four groups: underweight ($< 18.5 kg/m^2$), normal weight ($18.5-23.9 kg/m^2$), overweight ($24-27.9 kg/m^2$), and obese ($\ge 28 kg/m^2$).
- Vital capacity (VC): Vital capacity was measured using a calibrated spirometer (specific model and manufacturer information) following standardized guidelines. Each participant performed three maximal inhalations and exhalations, and the highest value was recorded.
- 50-metre run: The 50-metre run test assessed participants’ speed and agility. Participants sprinted for 50 metres on a marked track, and the time taken was measured using an electronic timer accurate to milliseconds.
- Sit-and-reach: Flexibility was evaluated through the sit-and-reach test. Participants sat on the floor with their legs extended and reached forward as far as possible while keeping their knees straight. The distance reached was measured using a standardized measuring device.
- Standing long jump: Participants’ explosive leg power was measured using the standing long jump. Participants jumped forward from a standing position, and the distance covered was measured from the starting line to the point where the back of their heels landed.
- 1000/800-metre runs (males/females): This test evaluated participants’ aerobic endurance. Male/female participants ran a distance of 1000/800 metres on a track, and the time taken was recorded.
- Pull-ups (males): Upper body strength was assessed by the number of pull-ups completed by male participants. Each participant performed as many pull-ups as possible with proper form.
- Sit-ups (females): Female participants’ core strength was evaluated through the sit-ups test. Participants performed as many sit-ups as possible within a fixed time frame.

## Introduction to Wannan Medical College

According to government regulations, Wannan Medical College required students to return to campus and resume classes starting in September 2020. However, preventive measures such as restricted access to campus, maintaining social distance, wearing masks, and avoiding gatherings were still in place. Regular nucleic acid testing was conducted for all students and faculty members; if an individual student was found to be infected, they were isolated along with those who had close contact with them. Although this required a significant amount of manpower and resources, the majority of students remained free from COVID-19, and any isolated cases were promptly handled without spreading the virus to others. On December 7, 2022, the Chinese government announced a new set of ten measures for epidemic prevention and control [15], marking the end of the prolonged three-year pandemic situation. As a result, students gradually returned to their normal daily lives.

## Statistical analysis

The physical fitness test data were analysed using SPSS 27.0 and G-power software. Effect size estimates, specifically Cohen’s d, were calculated to quantify the magnitude of differences between groups for quantitative variables, enhancing our understanding of the practical significance of observed changes. Additionally, statistical power calculations were performed using G-power software to ensure that our sample size was sufficient to detect meaningful effects and to enhance the reliability of our findings. The level of significance, sample size, effect size, and degrees of freedom were used as input parameters for the power analysis. Quantitative variables are summarized using means and standard deviations, while qualitative variables are described using frequencies and percentages. To assess the normal distribution of the data, we conducted the Shapiro-Wilk test to examine the *p*-values associated with the test items where these items all followed a normal distribution. Repeated measures analysis of variance (ANOVA) was employed to examine the physical fitness test indicators for the two groups at three consecutive time points following their enrolment. In cases where Mauchly’s assumption of sphericity was violated, the Greenhouse-Geisser correction was applied. Pairwise comparisons were conducted using the Bonferroni method. Statistical significance was set at a significance level of $P<0.05$, and two-tailed hypothesis testing was used with a 95% confidence interval. For brevity, we will refer to the three time points as the first year (2017 for the control group and 2019 for the experimental group), the second year (2018 for the control group and 2020 for the experimental group), and the third year (2019 for the control group and 2021 for the experimental group) after enrolment for both groups.

## Results

A total of 6580 students completed the physical fitness tests during the three years after their enrolment. The experimental group consisted of 3360 students (1490 males, 1870 females). The control group consisted of 3220 students (1326 males, 1894 females).

As shown in Table 1, for male students in both the experimental and control groups, all four weight categories shared the same trend. Specifically, the proportions of the three categories, including underweight, overweight, and obese students, consistently decreased, while the proportion of students with normal weight increased.

**Table 1:** The comparison of basic characteristics of male students in two groups

Group	Experimental group			Control group		
	Male(n=1490)			Male(n=1326)		
	1st year	2nd year	3rd year	1st year	2nd year	3rd year
Age(years)[n(%)]						
$\le 17$	115(7.71)	8(0.54)	0(0)	81(6.11)	5(0.38)	0(0)
18	727(48.79)	107(7.18)	8(0.54)	564(42.53)	76(5.73)	5(0.38)
19	494(33.15)	727(48.79)	107(7.18)	451(34.01)	564(42.53)	76(5.73)
20	124(8.32)	494(33.15)	727(48.79)	182(13.73)	451(34.01)	564(42.53)
21	26(1.74)	124(8.32)	494(33.15)	34(2.56)	182(13.73)	451(34.01)
$\ge 22$	4(0.27)	30(2.01)	154(10.34)	14(1.06)	48(3.62)	230(17.35)
Weight status [n(%)]						
Underweight	194(13.02)	171(11.48)	132(8.86)	156(11.76)	135(10.18)	128(9.65)
Normal	851(57.11)	901(60.47)	968(64.97)	807(60.86)	867(65.38)	877(66.14)
Overweight	315(21.14)	291(19.53)	282(18.93)	248(18.70)	219(16.52)	217(16.37)
Obesity	130(8.72)	127(8.52)	108(7.25)	115(8.67)	105(7.92)	104(7.84)

As shown in Table 2, for female students in both the experimental and control groups, the proportion who were underweight increased in the second year, followed by a rebound in the third year. The proportion of students of normal weight remained relatively stable. The proportion of obese students decreased in the second year and remained relatively unchanged the following year. Notably, the proportion of overweight in both the experimental and control groups decreased in the second year. However, in the third year, it remained relatively stable in the experimental group but increased in the control group.

**Table 2:** The comparison of basic characteristics of female students in two groups

Group	Experimental group			Control group		
	Female(n=1870)			Female(n=1894)		
	1st year	2nd year	3rd year	1st year	2nd year	3rd year
Age(years)[n(%)]						
$\le 17$	185(9.89)	17(0.91)	4(0.21)	208(10.98)	17(0.90)	2(0.11)
18	949(50.75)	168(8.98)	13(0.70)	859(45.35)	191(10.08)	15(0.79)
19	560(29.95)	949(50.75)	168(8.98)	581(30.68)	859(45.35)	191(10.08)
20	142(7.59)	560(29.95)	949(50.75)	192(10.14)	581(30.68)	859(45.35)
21	26(1.39)	142(7.59)	560(29.95)	40(2.11)	192(10.13)	581(30.68)
$\ge 22$	8(0.43)	34(1.82)	176(9.41)	14(0.74)	54(2.85)	246(12.99)
Weight status [n(%)]						
Underweight	367(19.63)	436(23.32)	392(20.96)	288(15.21)	409(21.59)	378(19.96)
Normal	1251(66.90)	1238(66.20)	1280(68.45)	1359(71.75)	1301(68.69)	1310(69.17)
Overweight	181(9.19)	141(7.54)	146(7.81)	189(9.98)	147(7.76)	169(8.92)
Obesity	71(3.80)	55(2.94)	52(2.78)	58(3.06)	37(1.95)	37(1.95)

**Fig. 2:**
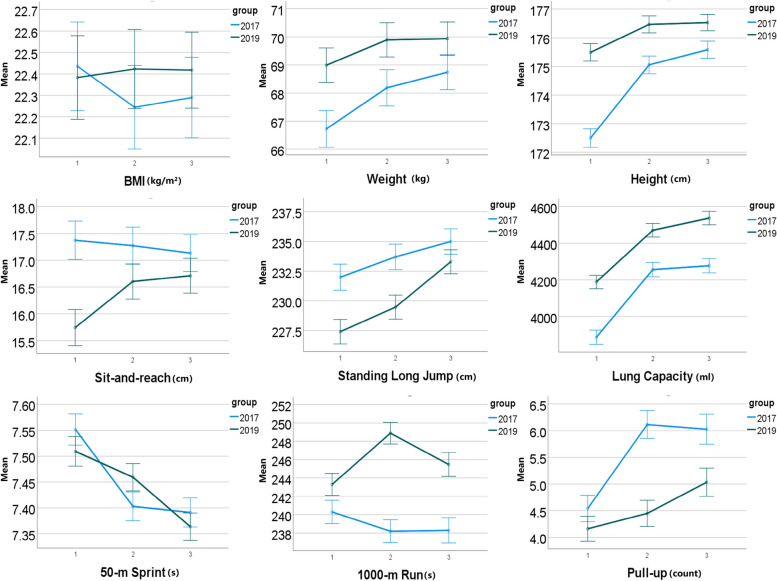
The average and 95% CI of items of the physical fitness tests on male students in control group (2017) and experimental group (2019) from the first year to the third year after their enrolment

**Fig. 3:**
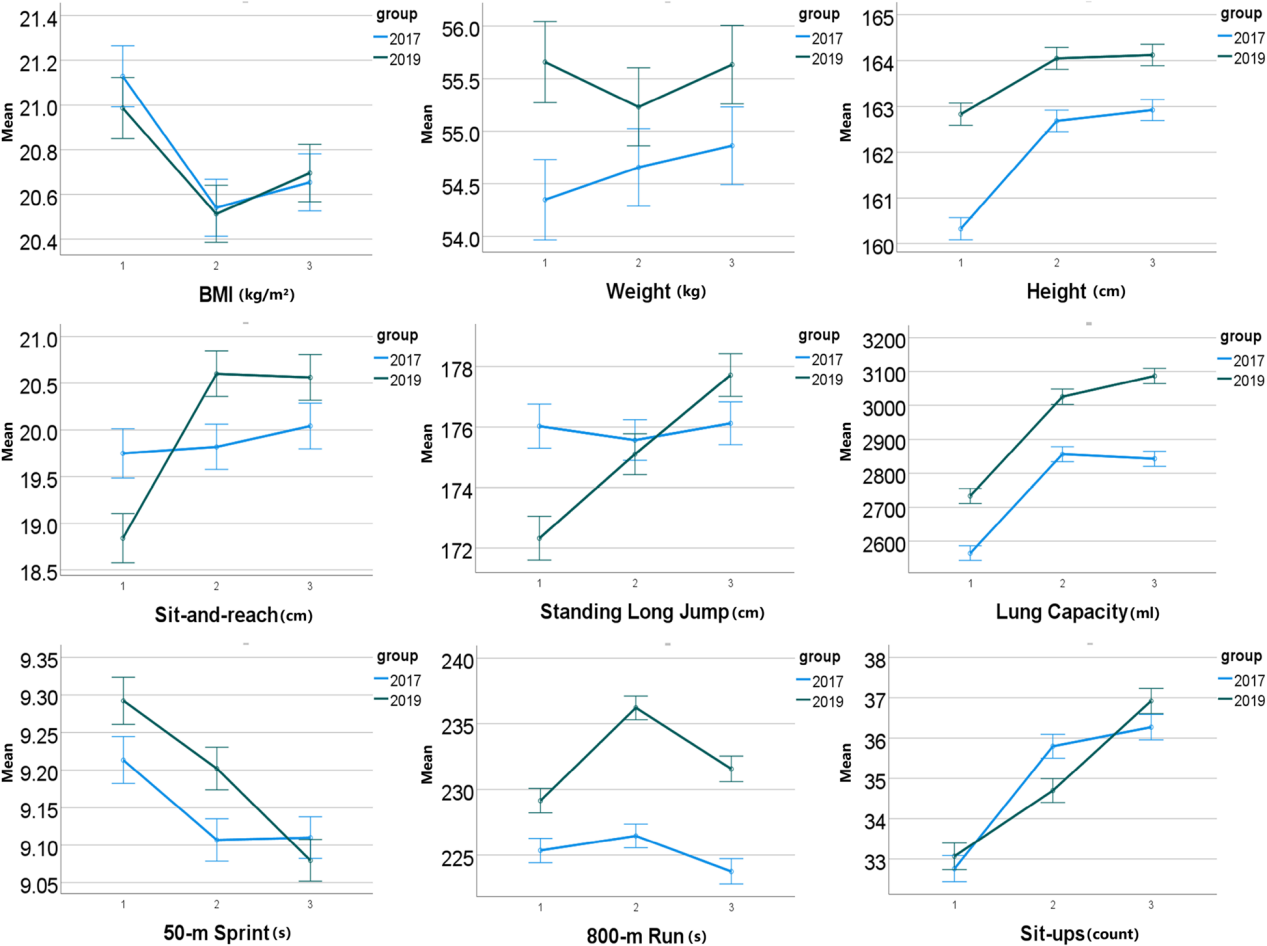
The average and 95% CI of items of the physical fitness tests on female students in control group (2017) and experimental group (2019) from the first year to the third year after their enrolment

To validate our hypothesis that home isolation environment has an adverse impact on the physical fitness development of university students, we examined the data from Tables 3 and 4 along with Figs. 2 and 3. In the male group, we observed significant trends between the experimental and control groups. For the 1000-metre run, there was a notable decrease in performance from the first to the second year, with the time increasing from 243 seconds to 249 seconds, indicating a decrease of 6 seconds ($P < 0.05$, effect size $d = 0.46$). However, in the third year, the 1000-metre run time decreased from 249 seconds to 245 seconds, resulting in an improvement of 4 seconds ($P < 0.05$, effect size $d = 0.28$). The average vital capacity scores exhibited significant improvements from the first to the second year, rising from 4189 milliliters to 4471 milliliters ($P < 0.05$, effect size $d = 0.30$). The trend continued in the third year, further increasing to 4538 milliliters ($P < 0.05$, effect size $d = 0.36$).

**Table 3:** The comparison of physical fitness indicators of male students in two groups

Group	Experimental group			Control group		
	Mean (SD)(n=1490)			Mean (SD)(n=1326)		
	1st year	2nd year	3rd year	1st year	2nd year	3rd year
Vital Capacity (ml)	4189(790)*	4471(762)*	4538(729)*	3887(648)	4256(688)	4277(720)
50-metre run (s)	7.5(0.5)*	7.5(0.5)*	7.4(0.5)	7.6(0.6)	7.4(0.5)	7.4(0.5)
Sit-and-reach (cm)	15.7(6.3)*	16.6(6.5)	16.7(6.2)	17.4(7.0)	17.3(6.4)	17.1(6.6)
Standing long jump (cm)	227(20)*	229(20)*	233(20)*	232(21)	234(20)	235(20)
1000-metre run (s)	243(25)*	249(26)*	245(28)*	240(22)	238(20)	238(23)
Pull-ups (count)	4(4)*	4(5)*	5(5)*	5(5)	6(5)	6(5)

* denotes *P*-values are less than 0.05

**Table 4:** The comparison of physical fitness indicators of female students in two groups

Group	Experimental group			Control group		
	Mean (SD)(n=1870)			Mean (SD)(n=1894)		
	1st year	2nd year	3rd year	1st year	2nd year	3rd year
Vital Capacity (ml)	2733(519)*	3025(517)*	3087(509)*	2564(455)	2856(473)	2843(484)
50-metre run (s)	9.3(0.7)*	9.2(0.6)*	9.1(0.6)	9.2(0.7)	9.1(0.6)	9.1(0.6)
Sit-and-reach (cm)	18.8(5.5)*	20.6(5.5)*	20.6(5.3)	19.8(6.2)	19.8(5.2)	20.0(5.5)
Standing long jump (cm)	172(16)*	175(15)	177(16)	176(16)	176(15)	176(15)
800-metre run (s)	229(22)*	236(22)*	232(22)*	225(19)	226(18)	224(20)
Sit-ups (count)	33(8)	35(7)*	37(7)*	33(7)	36(6)	36(7)

* denotes *P*-values are less than 0.05

Among female participants, we observed significant changes in the sit-and-reach test scores between the experimental and control groups. The average score increased from 18.8 centimeters in the first year to 20.6 centimeters in the second year ($P < 0.05$, effect size $d = 0.32$), but there was no significant difference in the third year ($P > 0.05$). For the 800-metre run, the average time increased from 229 seconds in the first year to 236 seconds in the second year ($P < 0.05$, effect size $d = 0.33$). However, from the second year to the third year, the average time decreased from 236 seconds to 232 seconds ($P < 0.05$, effect size $d = 0.21$). Furthermore, in the sit-ups test, we observed consistent improvements in scores. The average score increased from 33 counts in the first year to 35 counts in the second year ($P < 0.05$, effect size $d = 0.23$), and further increased to 37 counts in the third year ($P < 0.05$, effect size $d = 0.33$). These findings suggest that the home isolation environment has a notable impact on the scores of certain physical fitness test items.

Moreover, we calculated the statistical power of the conducted tests, with all exceeding 0.80, implying a high likelihood of detecting significant effects. This underscores the study’s ability to discern meaningful differences between the control and experimental groups.

## Discussion

This study aimed to assess the influence of home isolation during the pandemic on college students’ physical fitness development and examine changes in physical fitness test results. Conducted in Anhui Province, China, the research focused on students enrolled in 2017 (control group) and 2019 (experimental group), evaluating their performance in physical fitness tests over three years. While students in the experimental group showed decreased 1000-metre and 800-metre run scores following home isolation, their sit-and-reach scores improved. Notably, these trends aligned with previous findings [14].

Our research addressed the gap in understanding pandemic-related home isolation effects on college students’ physical fitness, revealing a specific impact on endurance and flexibility. The pandemic and ensuing isolation prompted reduced physical activity and increased sedentary behavior among students, affecting both physical and mental health [8].

Therefore, analyzing changes in various physical fitness test items is essential to comprehend home isolation’s effects on college students’ physical fitness. In terms of body composition, students’ heights displayed normal developmental trends over time, unaffected by isolation. Female students from the experimental group exhibited decreased body weight following home isolation, likely due to heightened activity levels during the pandemic [16]. This aligns with studies indicating female students’ increased concern for body image during isolation [17].

Regarding BMI, we observed a slight increase in the BMI index of male students in the experimental group after home isolation, while a more substantial decrease was observed among male students in the control group. We speculate that this could be attributed to the impact of home isolation caused by the pandemic. However, in the third years, there were no significant changes in the BMI indices of either group of male students. We believe that students can engage in normal exercise after returning to campus, thus mitigating the impact on the development of male BMI indices. As for female students, we observed very similar trends in BMI index development between the two groups, indicating that the impact of home isolation on BMI indices for female university students was relatively minor.

Further analysis of fitness test scores showed that vital capacity remained unaffected, suggesting the isolation environment had minimal influence. For muscle strength and explosiveness assessments (50-metre run and standing long jump), female students in the experimental group showed improvement. In contrast, male students exhibited no significant impact on standing long jump. The 50-metre run scores of both genders improved initially, but only the experimental group’s female students sustained upward trends, suggesting maintained exercise habits.

In the sit-and-reach test, we observed a significant improvement in flexibility for students in the experimental group after home isolation. We speculate that this might be due to students engaging in low-space-demanding exercises such as yoga, tai chi, and TikTok fitness routines during home isolation, promoting notable gains in flexibility. However, in the third year, students’ sit-and-reach scores tended to stabilize upon returning to campus, indicating a lack of targeted flexibility training in the school setting.

In the 1000-metre and 800-metre run tests, compared to the control group, the scores of students in the experimental group showed a significant decrease after one year of home isolation, but in the third year, students’ performance improved significantly, especially for female students. This indicates that home isolation had a negative impact on students’ endurance fitness. We speculate that the limitations imposed by home isolation restricted students from engaging in regular endurance training such as running. However, upon returning to campus, students’ scores for the middle- and long-distance run tests improved notably. This improvement can be attributed to the availability of space for long-distance aerobic running and slow jogging upon returning to campus.

Regarding the pull-ups (male) test, we found that the improvement in scores of male students in the experimental group after home isolation was not as significant as that in the control group. This suggests that male students might have overlooked upper body strength exercises during the isolation period. However, after returning to campus, the scores of the pull-ups test for male students in the experimental group began to improve, confirming our speculation. This result suggests that in future home isolation settings, attention should be paid to upper body strength exercises for male students, such as push-ups and similar exercises.

In the sit-ups (female) test, both groups of students exhibited similar trends in scores after home isolation. We speculate that this might be consistent with Fearnbach’s findings, indicating that using home exercise equipment to maintain physical activity levels during the pandemic was a common factor [18]. We believe that students engaged in a certain degree of exercise during home isolation, which helped maintain scores in this particular test.

Our study results indicate that after home isolation, there was a significant decrease in the middle- and long-distance run scores of students in the experimental group, a significant improvement in flexibility, improvement in standing long jump scores for female students, and no significant progress in pull-ups scores for male students. The trends in other test items were similar to those in the control group. Upon returning to campus two years after enrollment, i.e., in the third year, we observed a recovery in middle- and long-distance run scores, stable sit-and-reach scores, and an improvement in pull-ups scores for male students. This might be attributed to the space constraints of middle- and long-distance running, as well as the ability to conduct flexibility and strength training in smaller spaces. Therefore, we recommend focusing on muscle strength and endurance training during home isolation periods, such as using High-Intensity Interval Training (HIIT), TikTok fitness routines, stationary running, and shuttle running [19, 20]. Additionally, in school physical education, there should be an emphasis on flexibility training for students, such as practicing tai chi, whole-body vibration exercises, and similar activities [21, 22]. Furthermore, attention should not be disregarded on upper body strength training for male students, such as performing push-ups and similar exercises.

This study provided an important theoretical basis for improving the physical education curriculum system in higher education. Furthermore, it has practical value in guiding students’ physical exercise directions during home isolation in response to future public health emergencies to minimize the impact on students’ physical health. The strengths of this study lie in its large sample size and the use of a longitudinal repeated measurement design. However, there are two limitations in this study. First, the test time and population of the two groups were different due to the global impact of the pandemic, making it impossible to find two sample groups from the same time point for this study. Moreover, the two groups consisted of different individuals, which may introduce biases associated with variations in lifestyle habits and exercise routines. Second, the majority of the samples in this study were from Anhui Province, China, and therefore, the results may not be generalizable to different cultures and social backgrounds. In the future, it would be desirable to have a larger sample size that is more geographically diverse to study the impact of isolation environments on college students.

## Conclusion

In summary, our study offers valuable insights into the effects of pandemic-induced isolation environments on college students’ physical fitness. It emphasizes the need to address the impact on college students’ physical activity levels, endurance, and flexibility in future isolation environments. Moreover, our findings imply the importance of enhancing flexibility training and muscle strength training in school physical education, as indicated by the performances in sit-and-reach, as well as the 1000/800-metre tests, after students returned to campus in the third year after their enrolment.

## Data Availability

The datasets used and/or analysed during the current study are available from the corresponding author on reasonable request.

## Abbreviations

BMI: Body mass index
VC: Vital Capacity
HIIT: High-intensity interval training
